# BHGNN-RT: Capturing bidirectionality and network heterogeneity in graphs

**DOI:** 10.1371/journal.pone.0326756

**Published:** 2025-07-01

**Authors:** Xiyang Sun, Fumiyasu Komaki

**Affiliations:** 1 Mathematical Informatics Collaboration Unit, RIKEN Center for Brain Science, Wako, Saitama, Japan; 2 Department of Mathematical Informatics, The University of Tokyo, Bunkyo-ku, Tokyo, Japan; National University of Defense Technology, CHINA

## Abstract

Graph neural networks (GNNs) have shown great promise for representation learning on complex graph-structured data, but existing models often fall short when applied to directed heterogeneous graphs. In this study, we proposed a novel embedding method, a bidirectional heterogeneous graph neural network with random teleport (BHGNN-RT) that leverages the bidirectional message-passing process and network heterogeneity, for directed heterogeneous graphs. Our method captures both incoming and outgoing message flows, integrates heterogeneous edge types through relation-specific transformations, and introduces a teleportation mechanism to mitigate the oversmoothing effect in deep GNNs. Extensive experiments were conducted on various datasets to verify the efficacy and efficiency of BHGNN-RT. BHGNN-RT consistently outperforms state-of-the-art baselines, achieving up to 11.5% improvement in classification accuracy and 19.3% in entity clustering. Additional analyses confirm that optimizing message components, model layer and teleportation proportion further enhances the model performance. These results demonstrate the effectiveness and robustness of BHGNN-RT in capturing structural, directional information in directed heterogeneous graphs.

## Introduction

Graphs are a natural and powerful abstraction for representing complex systems in the real world, including citation networks, social networks, and the World Wide Web [1–7]. They offer a flexible framework for modeling entities and their relationships, making them well-suited for capturing intricate structural dependencies [8]. However, graph-structured data are often high-dimensional, sparse, and non-Euclidean, which makes their analysis particularly challenging [9]. To address this, Graph Neural Networks (GNNs) have emerged as a powerful graph representation learning method designed for such graph data and have attracted considerable research attention [10,11]. Traditional GNNs focus on individual nodes to generate a vector representation or an embedding for each node, such that two nodes “close” in the graph have similar vector representations in a low-dimensional space [12,13]. Recently, many variants of GNNs have achieved superior performances in network analysis, including node classification [14], graph classification, link prediction [15], and recommendations [16,17]. Examples include spectral graph convolutional neural networks [18–20], message-passing algorithms [21] and recurrent graph neural networks [22]. Among them, message-passing frameworks have received particular attention because of their flexibility and empirical effectiveness [19,23].

Graph structures and topological characteristics play a critical role in influencing the effectiveness of inference and learning in graph-based models [9,24,25]. Preserving these structural properties is essential for accurate graph representation learning. However, existing GNNs, particularly spectral-based GNNs, are primarily designed for undirected graphs and often overlook directionality [8,15,26]. In contrast, most real-world graphs are inherently directed. For example, in a citation network, newer papers may cite older ones, but not vice versa. This asymmetry between incoming and outgoing connections carries distinct semantic meanings and relational dynamics. Recent models, such as Directed graph convolutional network (DGCN) [8], Node Embedding Respecting Directionality (NERD) [27], Message Passing Attention network for Document understanding (MPAD) [28], and Directed Graph Neural Network (Dir-GCN) [29], attempted to extend GNNs to directed graphs. While these methods introduce direction-aware components like incoming message aggregation or second-order proximity, they often fail to fully capture the asymmetry between edge directions. Effectively integrating both incoming and outgoing information can yield more expressive node representations and is particularly crucial for modeling the functional roles and semantic context within directed graphs.

Another well-known limitation in graph neural networks is the issue of over-smoothing, where node embeddings become indistinguishable as the number of layers increases [25]. Theoretically, the message-passing process of *k* iterations takes advantage of a subtree structure with height *k* rooted at each node. Such schemes can generalize the Weisfeiler-Lehman graph isomorphism test to learn the distribution and topology of node features in the neighborhood simultaneously [14,30]. However, increasing the depth often degrades performance due to over-smoothing. For example, previous work indicated that the best performance of a SOTA model, the graph convolutional network (GCN), is achieved with a 2-layer structure [19]. Their embedding results converged to the random walk’s limit distribution as the layer number increased [19,23]. This phenomenon has also been reported in other GNN variants [30–32], limiting their ability to exploit long-range dependencies. In principle, deeper versions of GCN perform worse, although they have access to more information. The limitation of GNN layer configuration strongly restrains the expressivity of node neighborhoods with high path lengths. To mitigate this, we incorporate a random teleportation mechanism into our model, which injects stochasticity during embedding updates and preserves node-level distinctions [23,33]. By optimizing the teleportation proportion, our approach balances local and global information flow, helping to maintain expressive node representations even in deeper networks.

Existing GNN models often neglect the asymmetry of edge directions and the semantic diversity of node and edge types, leading to suboptimal performance in complex network settings. Moreover, many GNNs suffer from over-smoothing in deeper architectures, which limits their ability to capture long-range dependencies. To address these gaps, we propose a novel model: the Bidirectional Heterogeneous Graph Neural Network with Random Teleportation (BHGNN-RT). BHGNN-RT is beneficial to capture the network characteristics, including the bidirectional message-passing pathways and network heterogeneity. With random teleport, it can also mitigate oversmoothing and prevent information from stagnating in poorly connected nodes. To validate the effectiveness of BHGNN-RT, we conducted extensive experiments on benchmark datasets while comparing with the benchmark algorithms. Our results demonstrate that BHGNN-RT consistently outperforms existing methods, achieving state-of-the-art performance. We further analyze the impact of key components, including directional message integration, teleportation proportion, and network depth. Overall, this study contributes a unified framework for effectively learning on directed heterogeneous graphs and provides practical insights into the design of robust GNNs for complex networked systems.

## Related work

**GNNs for directed graphs.** Graph Neural Networks have traditionally been designed for undirected graphs, often overlooking the inherent directionality of many real-world networks [10,34]. Recognizing this limitation, recent research has focused on developing GNN architectures that effectively incorporate edge directionality to enhance learning on directed graphs [27,35]. Several models have been proposed to generalize spectral convolutions for directed graphs [8,36]. For instance, DGCN employed a generalized Laplacian via a personalized PageRank matrix and incorporates *k*-hop diffusion process [8]. MagNet utilized the magnetic Laplacian, a complex Hermitian matrix that encodes the magnitude and phase of graph connections, effectively capturing the directionality and structure of directed networks [37]. Additionally, adaptations of different message-passing frameworks have been proposed to enhance their applicability to directed networks. Gated Graph Sequence Neural Network (GGS-NN) employs modified gated recurrent units while aggregating messages only from output sequences [38]. Heterogeneous Directed Acyclic Graph (HetDAG) utilizes an attention-based directed graph learning module that fuses attributes and structures to search for an optimal graph representation between nodes [39]. Directed GNN (Dir-GNN) [29] is an example model designed to incorporate edge directionality into the message-passing process. It employs separate aggregations for incoming and outgoing edges by introducing asymmetry into the adjacency matrix to reflect directional relationships. Despite their advances, they typically focus on simple graph structures and do not empirically address the general functional forms for aggregating incoming and outgoing edges.

**GNNs for heterogeneous graphs.** Mathematically, a directed graph cannot simply be equivalently converted to an undirected relational network [29]. Relational Graph Convolutional Network (R-GCN) is designed to handle multi-relational graphs, focusing on link prediction and entity classification tasks [15]. Several papers deal with directed heterogeneous graphs by introducing inverse relations [40,41]. Dir-GCN can be treated as a Relational GCN applied to directed relational graphs with inverse edges [29]. While effective in modeling heterogeneity, most of the current approaches assume undirected edges, leaving the challenges posed by directed graphs largely unexplored.

## Network embedding strategy

### Problem formation

We formalized the problem on a directed heterogeneous graph 𝒢={𝒱,ℰ,𝒯,ℛ}, where 𝒱 represents the set of nodes (|𝒱|=N), ℰ denotes the set of edges (|ℰ|=M), 𝒯 is the set of node types, ℛ is the set of edge relations, and A is the adjacency matrix. In a heterogeneous graph, either the node types (𝒯) or the edge types (ℛ) are more than one (|𝒯|+|ℛ|>2). For a directed edge from node $v_{j}$ to node $v_{i}$, the matrix element $A_{ji}$ specifies its edge weight, where $A_{ji}=0$ if no edge exists. Node attributes are initialized as $X\in {\mathbb{R}}^{N\times f}$, where $x_{i}$ represents the feature vector of node $v_{i}$ and *f* is the feature dimension. Our goal is to develop an encoder to generate node embeddings $H\in {\mathbb{R}}^{N\times d}$ that effectively capture graph structure, heterogeneity, and directionality for downstream tasks such as node classification and clustering.

### Encoding bidirectionality and heterogeneity

To capture the structural and relational properties of directed heterogeneous graphs, we proposed a novel embedding strategy, named Bidirectional Heterogeneous Graph Neural Network with Random Teleport (BHGNN-RT), that integrates bidirectional message-passing and heterogeneity-aware mechanisms.

**Fig 1:**
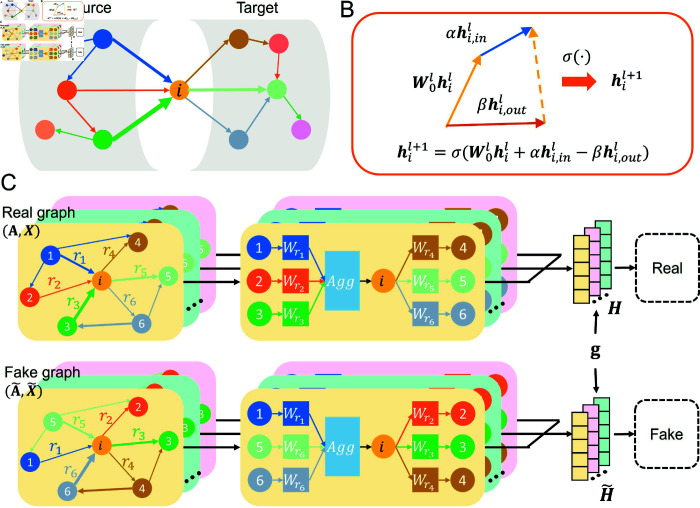
Illustration of the proposed BHGNN-RT framework. Panel A depicts an example of a directed heterogeneous graph, where an example node $v_{i}$ possesses distinct incoming and outgoing messages. B illustrates an updation function for node *i* during the message-passing process. Panel C describes how BHGNN-RT works for the clustering task.

For each node $v_{i}$, we distinguished the incoming and outgoing edges according to its corresponding source node set $N_{s}(v_{i})$ and target node sets $N_{t}(v_{i})$ (Fig 1A). This distinction is particularly important in the context of directed networks, such as social networks, and biological neural circuits, where afferent and efferent pathways normally deliver different kinds of messages [4,17,34,42]. Considering the edge relation r∈ℛ, the edge-dependent attention mechanism with learnable weight matrices $W_{r}$ was introduced to handle edge-level heterogeneity. Meanwhile, to reduce sensitivity to edge weight scaling, the weight of an edge from node $v_{j}$ to node $v_{i}$ was normalized by the coefficient $\frac{A_{ji}}{\sqrt{\sum_{k}A_{jk}}\sqrt{\sum_{k}A_{ki}}}$. For an unweighted graph, the normalization coefficient is $\frac{1}{\sqrt{deg^{-}(v_{i})}\sqrt{deg^{+}(v_{j})}}$, where $deg^{-}(\cdot )$ and $deg^{+}(\cdot )$ are the nodal in- and out-degrees, respectively.

In each iterable layer, the incoming messages with different edge relations to node $v_{i}$ were aggregated as follows:

$$h_{i,in}^{l}=\sum_{r\in ℛ}\sum_{j\in N_{s}^{r}(v_{i})}\frac{A_{ji}}{\sqrt{\sum_{k}A_{jk}}\sqrt{\sum_{k}A_{ki}}}W_{r}^{l}h_{j}^{l}$$ (1)

where $W_{r}^{l}$ represents a $d^{l+1}\;\times \;d^{l}$ weight matrix associated with edge type *r* and $h_{j}^{l}$ denotes the representation of node $v_{j}$ at *l*-th layer. Matrix $W_{r}^{l}$ shall be regularized by adopting basis decomposition [15].

Similarly, we consider the outgoing messages from node $v_{i}$ as the weighted summation of the hidden state $h_{i}^{l}$ from node $v_{i}$ itself instead of the hidden states of node neighborhood $N_{t}(v_{i})$. The outgoing messages are calculated as:

$$h_{i,out}^{l}=\sum_{r\in ℛ}\sum_{j\in N_{t}^{r}(v_{i})}\frac{A_{ij}}{\sqrt{\sum_{k}A_{ik}}\sqrt{\sum_{k}A_{kj}}}W_{r}^{l}h_{i}^{l}$$ (2)

Considering the distinct roles of incoming and outgoing messages, different coefficients should be assigned to both of them. Afterward, the nodal representation was updated based on the linear combination of its incoming and outgoing messages transformed on the *l*th layer, as shown in Fig 1B.

$${\bar{h}}_{i}^{l+1}=\sigma (W_{0}^{l}h_{i}^{l}+\alpha h_{i,in}^{l}-\beta h_{i,out}^{l})$$ (3)

where the activation function σ is a parametric rectified linear unit (PReLU), σ(·)=max(0,·)+amin(0,·) with a learnable parameter *a*. Hyperparameters α,β∈[0,1] control the contribution of different message components and are optimized during the training process.

Besides, we do not expect the message-passing process to be trapped in some specific nodes of the directed heterogeneous graph. Typically, nodes with strong self-loops or without outgoing edges easily absorb incoming messages and have little interaction with other nodes, where the message-passing process will not converge to the ideal embedding results. To overcome this problem, we draw inspiration from personalized PageRank [23,43] and introduce a teleport vector 1 into the aggregator function to denote the random pairwise connections in the graph. The teleport proportion is assigned with a probability γ∈[0,1]. The aggregator function is then finalized as:

$${\bar{h}}_{i}^{l+1}=\sigma (\gamma \frac{1}{N}+(1-\gamma )(W_{0}^{l}h_{i}^{l}+\alpha h_{i,in}^{l}-\beta h_{i,out}^{l}))$$ (4)

Afterward, the updated node embedding is normalized as $h_{i}^{l+1}=\frac{{\bar{h}}_{i}^{l+1}}{||h_{i}^{l+1}|{|}_{2}}$, where $\|h{\|}_{2}=\{\sum_{j=1}^{d}(h_{j}{)}^{2}{\}}^{1/2}$ is the standard Euclidean norm. After *L* layers of iteration, the final node embedding is produced as $H^{L}=(h_{1}^{L},\ldots ,h_{N}^{L})$.

### Objective functions

#### For node classification.

After stacking BHGNN-RT layers, we fed the output embedding $H^{L}\in {\mathbb{R}}^{N\times |𝒯|}$ with the activation function log_softmax to calculate the category scores $Z\in {\mathbb{R}}^{N\times |𝒯|}$, whose element is defined as $Z_{it}=\log(\frac{e^{H_{it}}}{\sum_{j=1}^{\left|𝒯\right|}e^{H_{ij}}})$.

The objective function was configured as a log-likelihood function that measures the gap between the ground truth and the predictions. A smaller gap indicates stronger consistency between them. We minimize the objective functions on all labeled data:

$$ℒ=-\frac{1}{N}\sum_{i}^{N}\sum_{t=1}^{\left|𝒯\right|}y_{it}Z_{it}$$ (5)

where *y*_*it*_ is the ground-truth label of node $v_{i}$ with node type *t*.

#### For node clustering.

To realize unsupervised clustering, we adopted the objective function inspired by the Deep Graph Infomax (DGI) to maximize the mutual information (MI) between node embeddings and a network summary [44]. The network representation is summarized as $g=f(H)=softmax(\frac{1}{N}\sum_{i=1}^{N}h_{i})$ by averaging all node embeddings in the graph. As a proxy for maximizing the mutual information between node-graph pairwise representations, a discriminator function is leveraged to measure its probabilistic score, which is defined as $S(h_{i},g)=\sigma (h_{i}^{T}M_{s}g)$. $M_{s}$ is a learnable scoring matrix, σ(·) is a sigmoid function and $h_{i}^{T}$ is the transpose of the node embedding $h_{i}$.

In parallel with the real graph, we generated a fake graph $\tilde{𝒢}$ by introducing row-wise shuffling of the adjacency matrix A and initial node features X (as Fig 1C). The row-wise shuffling followed a random permutation τ(i) for the sequence of each row (i=1,…,N). The fake graph was defined as $\tilde{𝒢}=(𝒱,\tilde{ℰ},𝒯,ℛ)$, where the edge set $\tilde{ℰ}$ consists of edges $\left(v_{\tau (i)},v_{j}\right)$ for $(v_{i},v_{j})\in ℰ$. The initial feature matrix was shuffled as $\tilde{X}=(x_{\tau (1)},...,x_{\tau (N)})$.

Concerning the set of original and fake graphs, the objective function is configured as

$$ℒ=\frac{1}{N}\sum_{i=1}^{N}(\log(S(h_{i},g))+\log(1-S({\tilde{h}}_{i},g))).$$ (6)

This log-likelihood value is derived from mutual information and assigns higher scores to positive embeddings and lower scores to negative ones. It encourages the embedding method to capture meaningful information shared across all nodes.

## Experimental setup

To evaluate the performance of our proposed BHGNN-RT model, we conducted extensive experiments across multiple benchmark datasets while comparing it with the SOTA algorithms. The experiments were designed to assess the effectiveness of the model in both node classification and unsupervised clustering tasks on directed heterogeneous graphs.

### Datasets

The experiments were conducted on six publicly available datasets, including Cora [45], Cora_ml [45], CiteSeer [46], CiteSeer_full [46], Amazon_CS [47], Amazon_photo [47]. These datasets are representative of directed heterogeneous graphs, where edges encode distinct relationships between nodes. Cora, Cora_ml, CiteSeer, and CiteSeer_full are classical citation graphs, where nodes represent articles and directed edges indicate citation relationships. Amazon_CS and Amazon_photo capture co-purchase relationships in an e-commerce context, where nodes represent products and edges denote products purchased together. Detailed statistics of the datasets are listed in S1 Table.

### Entity classification

To evaluate the model performance on node classification, we compared it with seven SOTA methods. These methods are divided into two categories: 1) spectral-based GNNs, such as ChebNet [18], GCN [19], simplifying GCN (SGC) [20], relational-GCN (R-GCN) [15]; 2) spatial-based GNNs, including GraphSAGE [14] and graph attention network (GAT) [21], directed GCN (Dir-GNN) [29]. The mechanisms of these baselines are described in the Appendix.

The experiments followed a consistent setup across all datasets to ensure fair evaluation. Nodes were randomly split into three subsets: 70% for training, 20% for validation, and 10% for testing. For training, we configured all models with hidden layer dimensions of 64 and tested the architecture of each model with layer depths ranging from 2 to 8. The configuration yielding the highest validation performance was used for evaluation. The weight matrices were initialized via the Glorot method and the Adam optimizer was adopted with a learning rate of 0.01. To ensure robustness, each experiment was repeated 10 times with different random seeds. The classification performance of each model was measured using accuracy and macro-F1 score. These metrics provided a comprehensive view of the model’s classification capabilities, accounting for both balanced and imbalanced datasets.

### Unsupervised clustering

The clustering task evaluates the ability of the proposed model to group similar nodes into clusters based on their embeddings. We utilized the embeddings generated by BHGNN-RT and compared its clustering performance with five baseline methods, including DGI [44], deep attentional embedded graph clustering (DAEGC) [48], Graph InfoClust (GIC) [49], Just Balance GNN (JBGNN) [50], and a variant of R-GCN [15]. Detailed descriptions of these baselines are provided in the Appendix.

To maintain consistency, all models were configured with hidden layer dimensions of 64 and output dimensions of 512. The appropriate numbers of model layers were chosen for each model based on their performance, shown as the red starred points in Fig 3. A maximum of 300 epochs was used for training and the Adam optimizer was employed with a learning rate of 0.001. The embeddings from GNN models were then served as input to the K-Means algorithm, which grouped nodes into *T* clusters. The experiments was repeated 10 times, and their performance was evaluated by comparing the predicted clusters with the ground truth labels. The clustering quality was evaluated using accuracy, normalized mutual information (NMI), and adjusted Rand index (ARI) [48,49]. NMI is a metric based on information theory, and ARI is treated as an accuracy metric that penalizes incorrect predictions.

**Fig 2:**
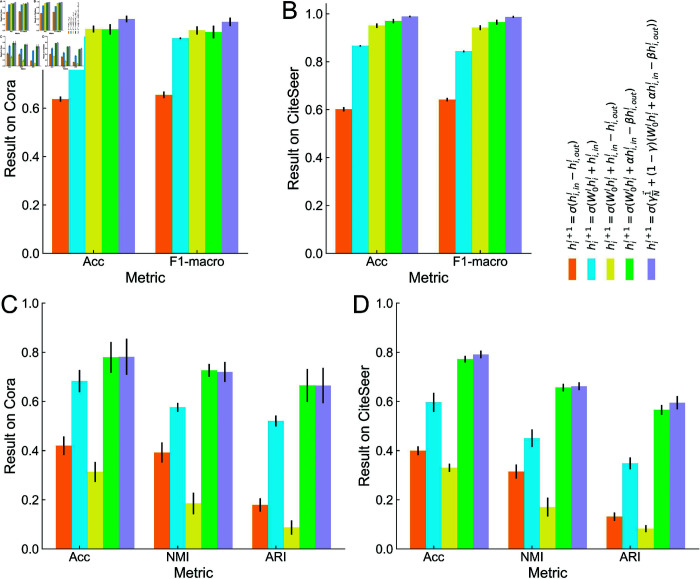
Evaluation of message components on model performance. Panels A, B depict the classification results, while Panels C, D display their clustering performance on Cora and CiteSeer. Each bar exhibits results with distinct aggregation functions, including 1) aggregation without nodal messages, 2) aggregation without outgoing messages, 3) aggregation with unweighted incoming and outgoing messages, 4) aggregation function as Eq 3, and 5) aggregation function as Eq 4.

We implemented all models and experiments using PyTorch 1.12.0 and CUDA toolkit 11.6. The experiments were conducted on a computer with a 20-core Intel i9-10900K CPU(@3.7 GHz), NVIDIA RTX A4000 GPU (16 GB memory), and 80 GB RAM. The code shall be made publicly available after the paper is accepted.

## Results

### Node classification

The classification experiments demonstrated that the proposed BHGNN-RT consistently outperforms SOTA baselines across all benchmark datasets. As summarized in Table 1, the classification accuracy of BHGNN-RT exceeds that of other models by margins ranging from 1.8% to 11.5%. The largest improvement was observed on the CiteSeer_full, where BHGNN-RT achieved an 11.5% higher accuracy compared to GAT (87.9±0.3%). A similar trend is evident for the macro-F1 metric, further highlighting the robustness of our approach. Meanwhile, BHGNN-RT consistently achieved better performance than the proposed model without random teleport (BHGNN), with improvements up to 4.3% on the Cora dataset. This demonstrates that the random teleport enhances the classification capability of our model.

**Table 1:** Node classification accuracy on benchmark datasets.

		ChebNet	GCN	SGC	GAT	GraphSAGE	Dir-GCN	R-GCN	BHGNN	BHGNN-RT
Cora	Acc	0.767	0.825	0.889	0.821	0.845	0.806	0.923	0.926	**0.969**
		±0.004	±0.002	±0.001	±0.008	±0.003	±0.003	±0.003	±0.021	± **0.014**
	Macro-F1	0.753	0.813	0.886	0.803	0.831	0.782	0.926	0.915	**0.957**
		±0.004	±0.002	±0.002	±0.011	±0.003	±0.003	±0.004	±0.026	± **0.017**
Cora_ml	Acc	0.831	0.816	0.822	0.809	0.836	0.869	0.906	0.976	**0.997**
		±0.006	±0.003	±0.004	±0.005	±0.005	±0.003	±0.003	±0.011	± **0.002**
	Macro-F1	0.828	0.803	0.804	0.798	0.814	0.860	0.898	0.973	**0.997**
		±0.007	±0.005	±0.006	±0.005	±0.005	±0.003	±0.004	±0.012	± **0.002**
CiteSeer	Acc	0.739	0.706	0.732	0.713	0.764	0.757	0.889	0.970	**0.989**
		±0.002	±0.003	±0.002	±0.010	±0.003	±0.002	±0.003	±0.009	± **0.003**
	Macro-F1	0.701	0.671	0.702	0.684	0.727	0.724	0.872	0.965	**0.987**
		±0.003	±0.003	±0.002	±0.011	±0.004	±0.002	±0.004	±0.010	± **0.004**
CiteSeer_full	Acc	0.803	0.873	0.850	0.879	0.834	0.840	0.857	0.988	**0.994**
		±0.003	±0.002	±0.008	±0.003	±0.007	±0.004	±0.002	±0.002	± **0.002**
	Macro-F1	0.805	0.874	0.850	0.880	0.836	0.841	0.858	0.989	**0.994**
		±0.003	±0.002	±0.009	±0.003	±0.007	±0.004	±0.002	±0.002	± **0.002**
Amazon_cs	Acc	0.840	0.881	0.905	0.900	0.885	0.900	0.963	0.984	**0.985**
		±0.016	±0.007	±0.003	±0.004	±0.006	±0.010	±0.002	±0.001	± **0.001**
	Macro-F1	0.769	0.858	0.886	0.897	0.860	0.873	0.960	**0.984**	**0.981**
		±0.044	±0.011	±0.004	±0.005	±0.009	±0.018	±0.003	± **0.003**	**±0.002**
Amazon_photo	Acc	0.915	0.943	0.937	0.940	0.939	0.942	0.974	0.985	**0.992**
		±0.017	±0.006	±0.002	±0.005	±0.008	±0.008	±0.002	±0.002	± **0.007**
	Macro-F1	0.892	0.932	0.927	0.930	0.927	0.928	0.973	0.983	**0.992**
		±0.017	±0.006	±0.002	±0.005	±0.008	±0.008	±0.002	±0.002	± **0.002**

### Node clustering

For clustering tasks, BHGNN-RT remains superior performance compared to other baselines across multiple datasets. Table 2 lists the highest clustering results over 10 runs for the proposed and baseline models, and the results with the mean and standard deviation are reported in S2 Table. Ten repetitions for comparative experiments with different random seeds are widely adopted in graph neural network researches and provide a reasonable estimation of performance variability while maintaining computational feasibility. Notably, on the CiteSeer dataset, BHGNN-RT outperformed the best baseline, GIC, by a substantial margin of 19.3% in accuracy. Improvements in NMI and ARI were also significant, ranging from 2.1% to 18.1% and 7.6% to 29.2%, respectively. It is promising that our proposed method allows each node stronger access to the structural properties of global connectivity.

**Table 2:** Clustering performance on benchmark datasets.

		K-Means	DGI	DAEGC	GIC	JBGNN	R-GCN-v	BHGNN	BHGNN-RT
	Acc	0.408	0.696	0.547	0.672	0.483	0.767	0.876	**0.889**
Cora	NMI	0.215	0.516	0.371	0.494	0.384	0.653	0.758	**0.790**
	ARI	0.124	0.470	0.324	0.400	0.266	0.559	0.764	**0.777**
	Acc	0.514	0.699	0.518	0.647	0.379	0.507	0.806	**0.815**
Cora_ml	NMI	0.330	0.512	0.364	0.485	0.254	0.467	**0.660**	**0.650**
	ARI	0.226	0.488	0.285	0.391	0.167	0.309	0.625	**0.633**
	Acc	0.440	0.607	0.602	0.625	0.468	0.612	0.791	**0.818**
CiteSeer	NMI	0.210	0.370	0.306	0.367	0.251	0.504	0.678	**0.685**
	ARI	0.158	0.338	0.309	0.348	0.235	0.343	0.600	**0.640**
	Acc	0.476	0.597	0.593	0.754	0.516	0.440	0.822	**0.852**
CiteSeer_full	NMI	0.344	0.482	0.331	0.506	0.317	0.280	0.671	**0.687**
	ARI	0.098	0.216	0.310	0.495	0.265	0.166	0.615	**0.665**
	Acc	0.231	0.207	0.542	0.470	0.377	0.721	0.758	**0.766**
Amazon_cs	NMI	0.120	0.029	0.413	0.461	0.397	0.772	**0.793**	**0.789**
	ARI	0.064	0.033	0.395	0.278	0.215	0.589	**0.665**	**0.658**
	Acc	0.275	0.236	0.651	0.572	0.513	0.879	**0.951**	**0.944**
Amazon_photo	NMI	0.143	0.044	0.545	0.527	0.424	0.830	**0.898**	**0.893**
	ARI	0.063	0.019	0.455	0.326	0.289	0.797	**0.913**	**0.905**

### Effects of message components

To analyze the contribution of individual message components, we performed ablation studies by modifying the aggregation functions in BHGNN-RT. In the traditional message-passing process, people mainly pay attention to the incoming messages [14,19,21,29]. Results in Fig 2 indicate that both nodal and outgoing messages play a critical role in graph representation learning, as evidenced by the comparison between configurations excluding nodal or outgoing messages. Interestingly, the inclusion of unweighted incoming and outgoing messages yielded good results in classification but underperformed in clustering tasks. We assume that this phenomenon occurs because the optimization of mutual information between node-graph representations affects the balance between incoming and outgoing messages when without ground truth. This discrepancy underscores the importance of optimizing the coefficients (α,β) of message components. Specifically, while the full integration improves predictive performance by capturing richer structural information, it can also increase model complexity due to the additional parameters introduced by relation-specific transformation matrices and the need to compute multiple aggregation pathways. Integrating all message components led to the best classification and clustering performance, demonstrating the efficacy of the proposed message-passing framework.

### Effects of model layers

**Fig 3:**
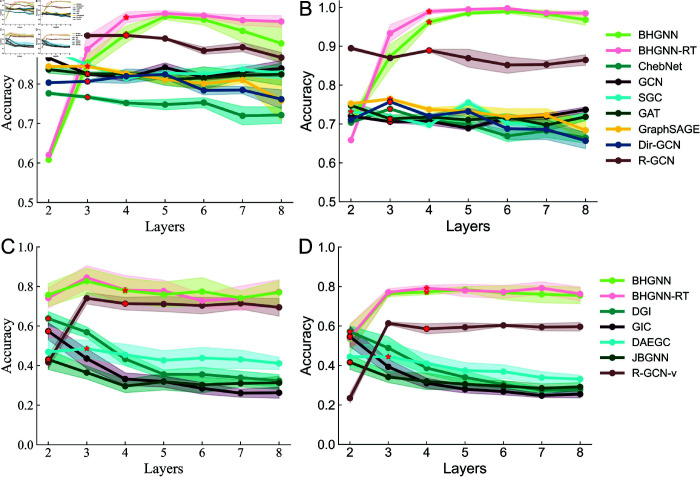
Effect of network layers for classification and clustering tasks. A and B exhibit classification results, while C and D show clustering results on Cora and CiteSeer. Legends in panels B and D indicate methods used for classification and clustering tasks, respectively. The configuration of model layers is marked as the red stars.

The impact of varying the number of network layers was also evaluated. Generally speaking, each node interacts with information from the *l*-hop neighborhood when stacking *l* GNN layers [51], leading to over-smoothing and overfitting [30,31]. As shown in Fig 3, the test accuracy for BHGNN and BHGNN-RT increases as the number of layers grows from 2 to 4. Unlike other baselines, which exhibit performance degradation due to over-smoothing with deeper layers, BHGNN-RT maintains stable performance beyond four layers across different datasets. This resilience highlights the model’s ability to effectively suppress over-smoothing.

For simplicity and computational efficiency, we configured BHGNN-RT with four layers for all experiments, as higher layer counts did not yield significant improvements. The appropriate layers of all models are configured as red stars in Fig 3. This configuration strikes a balance between performance and computational complexity.

### Visualization of embedding results

To provide a qualitative assessment of the learned embeddings, we applied the t-SNE method to visualize their clustering results across different datasets. The nodes are colored based on their labels in S1 Table. The clustering results were evaluated using silhouette scores (SIL), a metric to quantify the quality of the clusters generated.

**Fig 4:**
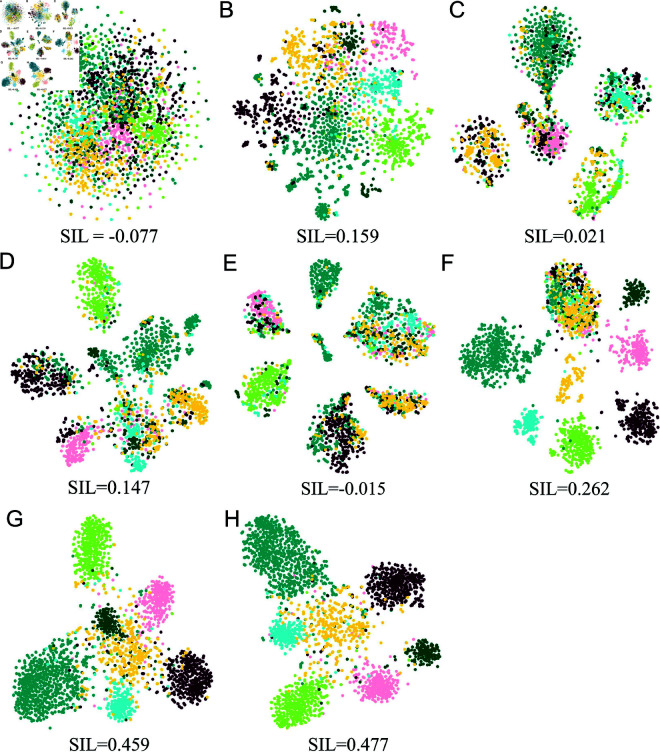
t-SNE visualization for clustering Cora datasets and the corresponding SIL scores. Individual panel depicts the results from different methods, including K-means (A), DGI (B), DAEGC (C), GIC (D), JBGNN (E), R-GCN-v (F), BHGNN (G), and BHGNN-RT (H).

**Fig 5:**
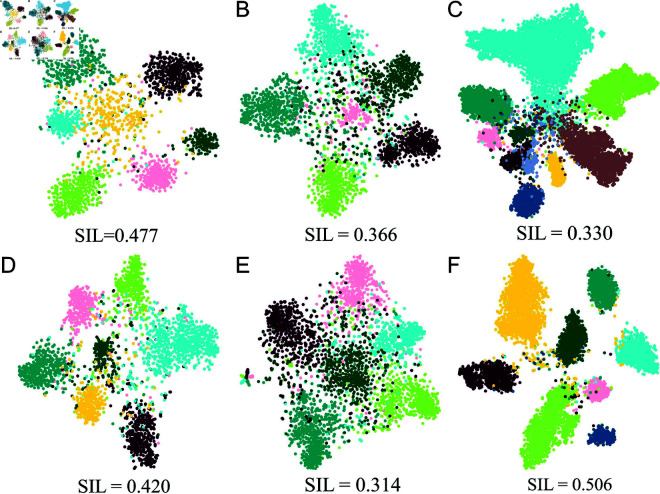
t-SNE plots for clustering different datasets via BHGNN-RT and relevant SIL scores. The datasets contain Cora (A), Cora_ml (D), CiteSeer (B), CiteSeer_full (E), Amazon_cs (C), and Amazon_photo (F).

As shown in Fig 4 and S1 Fig, BHGNN-RT achieves clearer clustering boundaries, separating nodes with the same labels into distinct groups. Among all methods, BHGNN-RT achieved the highest SIL score of 0.477 for Cora and 0.506 for Amazon dataset, indicating superior clustering quality. In addition, the clustering performance of BHGNN-RT was evaluated across diverse datasets (Fig 5), with consistent results observed. Particularly on the Amazon datasets, the method produced more distinct clusters, likely due to the higher average degrees in these graphs for stronger network connectivity density. In sum, our method improves unsupervised clustering quality when capturing more comprehensive nodal connectivity profiles and graph-level structural properties.

## Conclusion

In this study, we proposed BHGNN-RT, a novel graph neural network designed specifically for directed heterogeneous graphs. The model effectively incorporates bidirectional message-passing and accounts for network heterogeneity, ensuring high-quality graph representation learning. By optimizing the teleportation proportion, BHGNN-RT balances information from neighboring nodes and random connections, which significantly mitigates the over-smoothing issue prevalent in deep GNNs. Furthermore, the model is compatible with both unweighted and weighted graphs, making it versatile for a range of complex graph scenarios.

Extensive experiments were conducted to evaluate the effectiveness of the proposed BHGNN-RT model. The model achieved state-of-the-art performance across node classification and unsupervised clustering tasks, consistently outperforming existing baselines. Our method demonstrated notable improvements, particularly in capturing bidirectional edge semantics and preserving feature heterogeneity. This is further supported by the model’s superior performance in clustering tasks, where BHGNN-RT produced more distinct and meaningful node groupings. Beyond the quantitative results, we investigated the impact of model components, including the effects of message-passing configurations, the number of layers, and teleportation. Our analysis revealed that the inclusion of both nodal and outgoing messages contributes substantially to improved performance, while careful optimization of message coefficients and teleportation proportions further enhances the results. Notably, BHGNN-RT demonstrated superior clustering performance in graphs, producing more distinct and meaningful clustering patterns. The findings underscore the model’s ability to generalize effectively while addressing critical challenges in directed heterogeneous graphs.

Looking ahead, future research can explore extending BHGNN-RT to dynamic and temporal graphs, which introduce additional complexities such as time-evolving structures and edge dynamics. We also plan to investigate advanced combinations of node- and layer-wise aggregation functions to further enhance the model’s flexibility and adaptability to diverse graph types. These directions aim to solidify the applicability of BHGNN-RT in real-world scenarios and expand its scope to emerging challenges in graph representation learning.

## Supporting information

Programs. The python codes for our GNN model, experiments, and evaluation of performances are all in the BHGNN.ipynb file.

S1 Table Statistics of the datasets for directed heterogeneous graphs. The table lists the number of nodes, edges, node classes, edge relations, and the dimension of node features. The average degree measures the average number of edges per node in the graph.
(PNG)

S2 Table Clustering performance on benchmark datasets. This table records the average results and standard deviations for clustering performance on 10 runs. We configured the random_state in K-means as 0, in which case its results are the same across different runs. The best results are depicted in bold.
(PNG)

S1 Fig t-SNE visualization for clustering Amazon_photo datasets and the corresponding SIL scores. Individual panel depicts the results from different methods, including K-Means (A), DGI (B), DAEGC (C), GIC (D), JBGNN (E), R-GCN-v (F), BHGNN (G), and BHGNN-RT (H).
(JPG)
