# Supplementary figures and images for: BHGNN-RT: Capturing bidirectionality and network heterogeneity in graphs
Source: PLoS One. 2025 Jul 1;20(7):e0326756. doi: 10.1371/journal.pone.0326756 (PMC12212746; doi:10.1371/journal.pone.0326756)

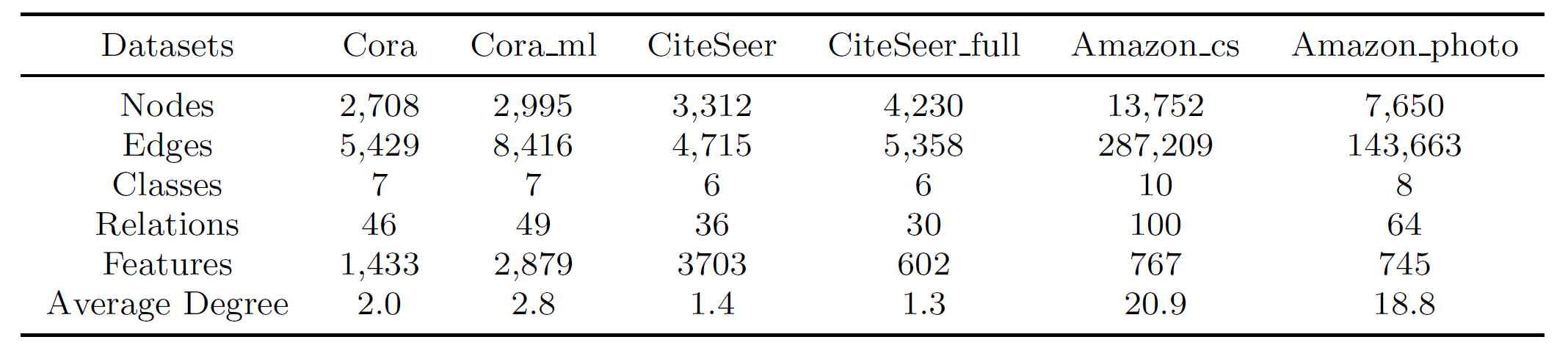

Supplement: S1 Table — The table lists the number of nodes, edges, node classes, edge relations, and the dimension of node features. The average degree measures the average number of edges per node in the graph. (PNG) [file pone.0326756.s001.png]

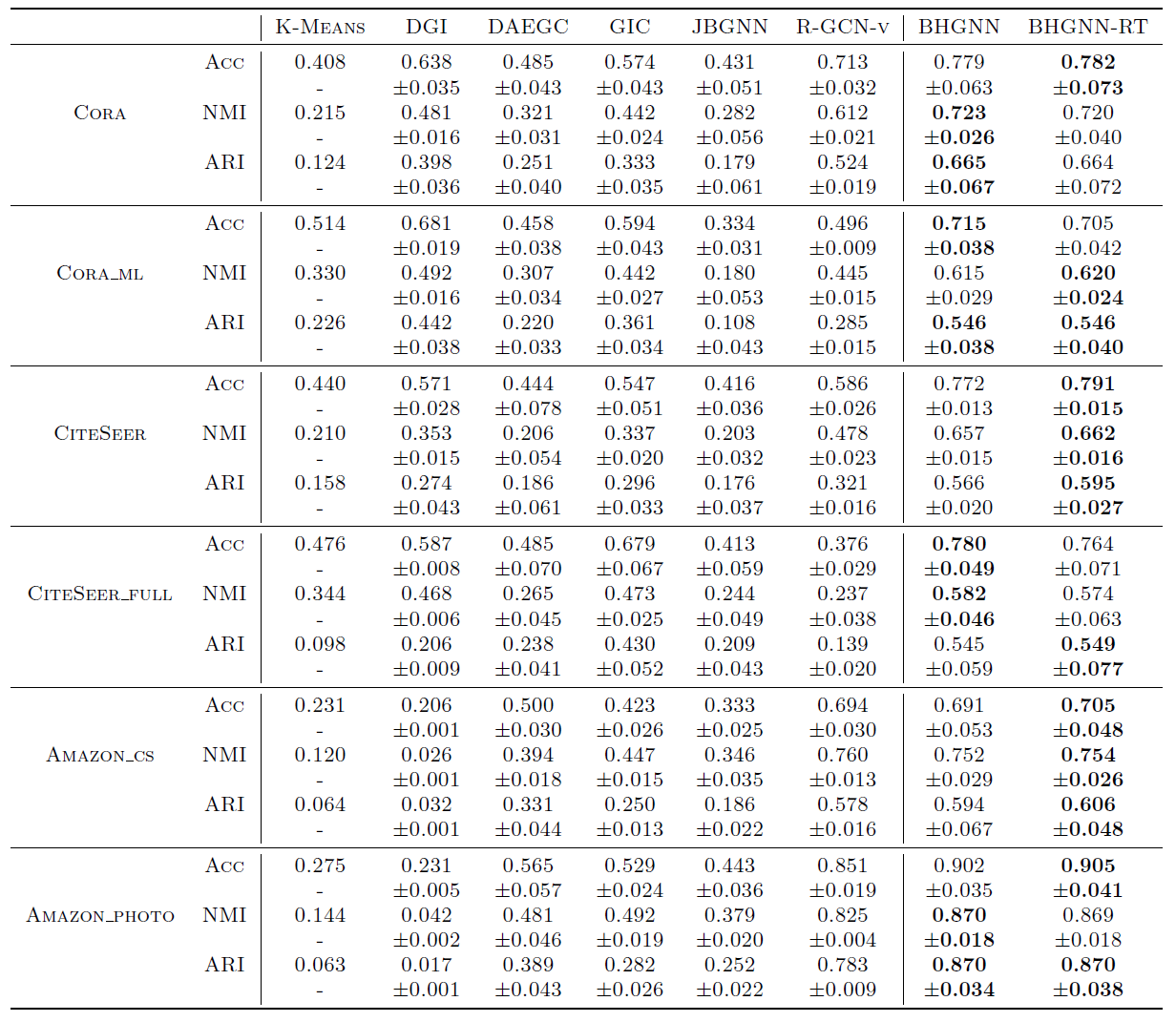

Supplement: S2 Table — This table records the average results and standard deviations for clustering performance on 10 runs. We configured the random_state in K-means as 0, in which case its results are the same across different runs. The best results are depicted in bold. (PNG) [file pone.0326756.s002.png]

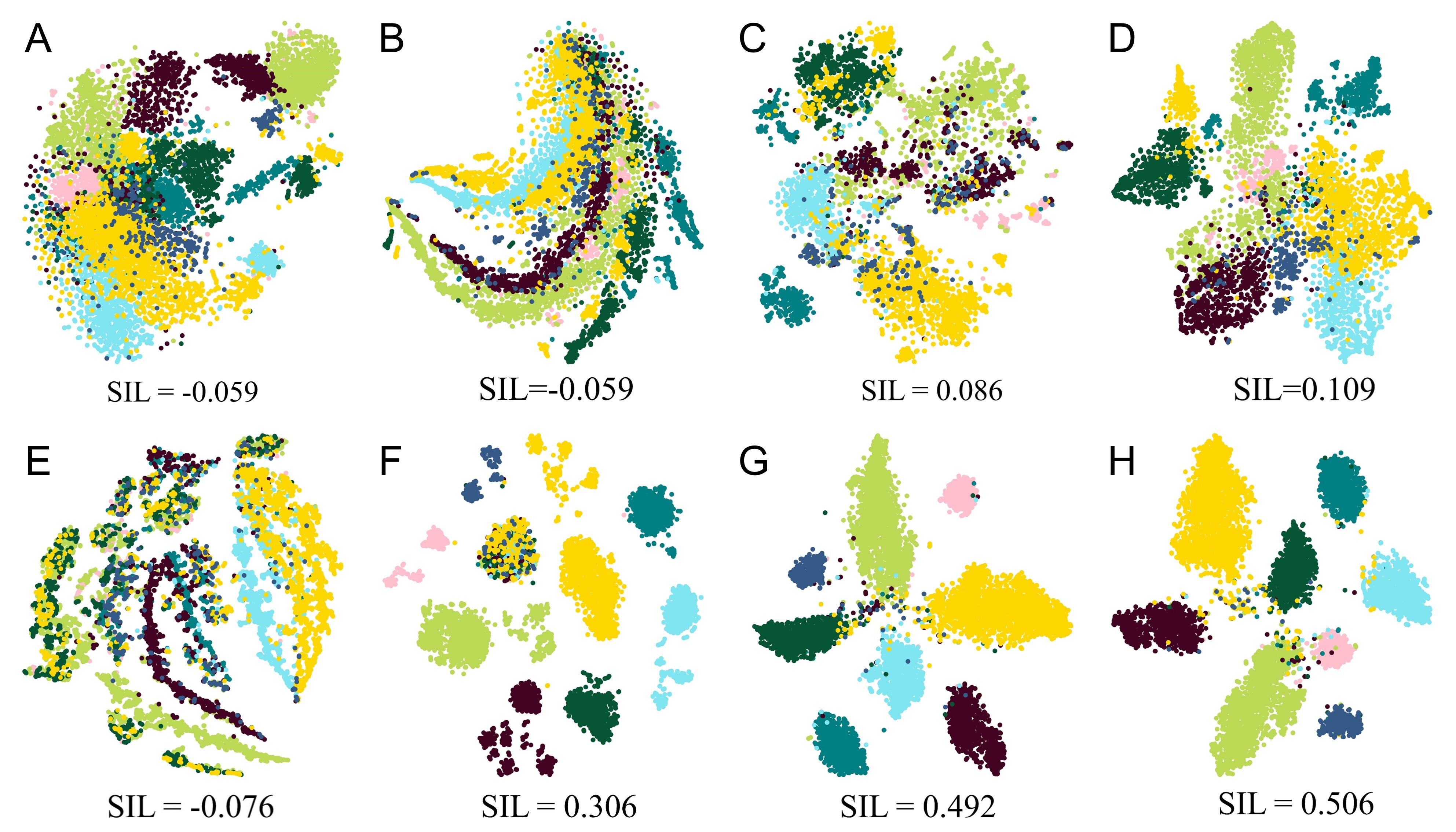

Supplement: S1 Fig — Individual panel depicts the results from different methods, including K-Means (A), DGI (B), DAEGC (C), GIC (D), JBGNN (E), R-GCN-v (F), BHGNN (G), and BHGNN-RT (H). (JPG) [file pone.0326756.s003.jpg]
